# Role of surface plasmon polaritons and other waves in the radiation of resonant optical dipole antennas

**DOI:** 10.1038/srep08456

**Published:** 2015-02-13

**Authors:** Hongwei Jia, Haitao Liu, Ying Zhong

**Affiliations:** 1 Key Laboratory of Optical Information Science and Technology, Ministry of Education, Institute of Modern Optics, Nankai University, Tianjin 300071, China; 2 State Key Laboratory of Precision Measuring Technology and Instruments, Tianjin University, Tianjin 300072, China

## Abstract

The radiation of an electric dipole emitter can be drastically enhanced if the emitter is placed in the nano-gap of a metallic dipole antenna. By assuming that only surface plasmon polaritons (SPPs) are excited on the antenna, we build up an intuitive pure-SPP model that is able to comprehensively predict the electromagnetic features of the antenna radiation, such as the total or radiative emission rate and the far-field radiation pattern. With the model we can distinguish the respective contributions from SPPs and from other surface waves to the antenna radiation. It is found that for antennas with long arms that support higher-order resonances, SPPs provide a dominant contribution to the antenna radiation, while for other cases, the contribution of surface waves other than SPPs should be considered. The model reveals an intuitive picture that the enhancement of the antenna radiation is due to surface waves that are resonantly excited on the two antenna arms and that are further coupled into the nano-gap or scattered into free space. From the model we can derive a phase-matching condition that predicts the antenna resonance and the resultant enhanced radiation. The model is helpful for a physical understanding and intuitive design of antenna devices.

Resonant optical nano-antennas are intensively studied in recent years due to their superior properties of generating strong electromagnetic field under far-field illuminations^1^,^2^,^3^,^4^,^5^,^6^ and reciprocally, enhancing the radiation of emitters such as molecules or quantum dots in the vicinity of antennas^7^,^8^,^9^,^10^,^11^,^12^,^13^,^14^,^15^,^16^,^17^,^18^,^19^. Plasmonic nano-antennas are widely used in enhanced Raman scattering spectroscopy^20^,^21^,^22^,^23^, nonlinear optical control^24^,^25^,^26^,^27^, and single-emitter fluorescence enhancement^9^,^10^,^11^,^15^,^16^,^17^,^18^,^28^. Much experimental and theoretical work has been devoted to achieve an understanding of the underlying physics of resonant nano-antennas for guiding the design of relevant devices. For a simple single-wire nano-antenna, it is described as a Fabry-Pérot resonator of surface plasmon polaritons (SPPs)^4^,^5^,^12^,^19^ for predicting the resonance frequency. The single-wire nano-antenna is also treated as an equivalent circuit composed of resistors, inductors and capacitors^29^,^30^,^31^, and radiation or scattering features such as the resonance frequency and the extinction spectrum can be predicted. Resonant dipole antennas, which are made of two metallic nano-wires separated by a nano-gap^1^, can achieve a much stronger enhancement effect than the single-wire antenna. Concepts of impedance and resistance are proposed for dipole antennas^32^,^33^,^34^,^35^ for reproducing quantities such as the resonance frequency, the quantum efficiency and the enhancement of field. The dipole antennas are also modelled as one-dimensional micro-cavities^34^,^36^, and the enhancement effect is attributed to the resonance of SPPs. In previous literatures, it is commonly believed that the enhancement effect of the antenna radiation or the associated near field is due to a resonant excitation of SPPs on the antenna arms^4^,^5^,^12^,^19^,^29^,^30^,^31^,^32^,^33^,^34^,^35^,^36^. This intuitive belief, which plays a central role in present antenna theories, will be checked at a quantitative level in the present work. In addition, the dynamical excitation and scattering process of SPPs in dipole antennas will be clarified.

In this work, we theoretically investigate the interaction between an electric dipole source and the dipole antenna. By considering the dynamical process that the SPP is first launched by the emitter in the nano-gap and then propagates along the antenna arms before further scattered by the arm end or the nano-gap, we build up a pure-SPP model that excludes the contribution from other surface waves to the antenna radiation. The model can comprehensively predict the electromagnetic features of the antenna radiation such as the emission rates and the far-field radiation pattern. Through the comparison between the prediction of the SPP model and fully-vectorial numerical data, we find that for dipole antennas with long arms that support higher-order resonances, the SPP imposes dominant impact on the radiation of the antenna. But for dipole antennas with short arms that support lower-order resonances, surface waves other than SPPs also contribute considerably to the antenna radiation. From the model we can derive a phase-matching condition for predicting the resonance of the antenna. Intuitive analysis is provided with the model for understanding the impact of different factors (such as the wavelength or the antenna geometrical parameters) on the antenna radiation.

## Fully-vectorial numerical method

**Figure 1:**
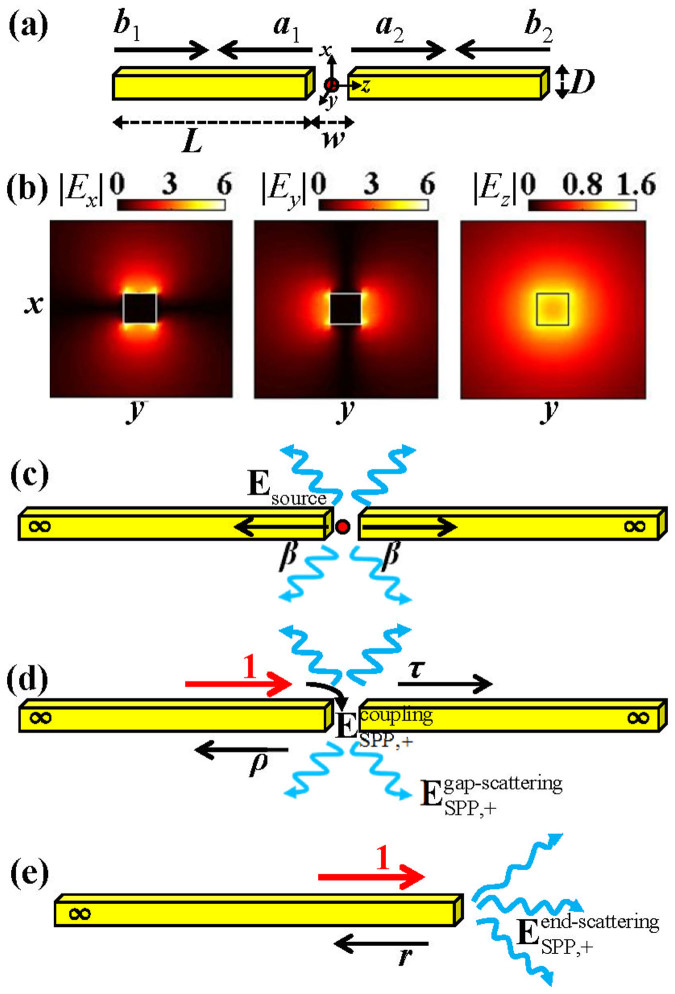
(a) Sketch of the dipole antenna. The antenna is composed of two arms of gold nano-wires separated by a nano-gap, and is illuminated by a *z*-polarized dipole source in the nano-gap. *a*_1_, *a*_2_, *b*_1_, *b*_2_ are the unknown coefficients of SPPs to be solved with the SPP model. (b) Distribution of the electric-field amplitude of the fundamental SPP mode on the *x*–*y* cross section (obtained for *D* = 40nm and *λ* = 1μm). (c)–(e) Scattering coefficients, *β*, *ρ*, *τ*, *r*, and fields, **E**_source_, 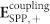, 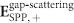 and 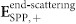 that appear in the equations of the model. (1 column)

The considered dipole antenna is sketched in Fig. 1(a), which is composed of two arms of gold nano-wires separated by a nano-gap (width *w*). A *z*-polarized electric dipole source, which represents the emitting molecules^8^,^9^,^10^,^11^,^14^,^15^,^17^,^18^ or quantum dots^7^,^13^,^19^, is put at the center of the nano-gap. The two arms have a square cross section (side length *D*) and have the same length *L*. The origin of the coordinate is set at the center of the gap. For simplicity, the antenna is put in air (refractive index *n_a_* = 1) without a substrate. The refractive index of gold (denoted by *n_m_*) for different wavelengths takes tabulated values from Ref. 37.

To obtain rigorous data of the radiation of the antenna, we use a fully-vectorial aperiodic Fourier modal method (a-FMM)^38^ [the calculation is performed with an in-house software, Liu, H. T., DIF code for modeling light diffraction in nanostructures (Nankai University, China, 2010)]. The a-FMM is a generalized version of the well-developed rigorous coupled wave analysis (RCWA)^39^,^40^. RCWA has been widely used in rigorous modeling of periodic electromagnetic systems. The electromagnetic field in transverse periodic directions is discretized upon Fourier basis and the frequency-domain Maxwell's equations are integrated analytically along the other propagation direction. By incorporating perfectly matched layers in the transverse directions to build up artificial periodicity, the a-FMM is then applied for aperiodic systems with the same algorithm of RCWA. Some details of the a-FMM can be found in the Supplementary Information.

The *z*-polarized electric point source in the nano-gap can be expressed as **J** = *δ*(*x*,*y*,*z*)**z**, with *δ* the Dirac function, and **z** the unit vector along the *z*-direction. The total emission rate (also called total decay rate) of the source is calculated with Γ_total_ = −Re[*E_z_*(0,0,0)]/2, where Re[*E_z_*(0,0,0)] is the real part of the *z*-component of electric field at the source position. The total emission rate of the dipole power further decays into non-radiative (e.g. loss as heat) and radiative (photon emission) channels. The radiative emission rate (denoted by Γ_rad_) is calculated with the integral 
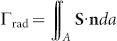
, where *A* is a closed surface encompassing the dipole antenna, **S** is the time-averaged Poynting vector, and **n** is the out-pointing normal vector on *A*.

## Pure-SPP model

Here we build up a pure-SPP model by considering the dynamical launching and multiple-scattering process of SPPs on the dipole antenna. Since the size of the cross section of the antenna is much smaller than the wavelength, only the fundamental SPP mode is bounded (field decaying to null at infinity in transversal *x*- and *y*-directions) and propagative (propagation constant being almost real) on the antenna^41^. The field distribution of the fundamental SPP mode on the *x*-*y* cross section is shown in Fig. 1(b). In the model only the fundamental SPP mode on the antenna is considered and all other surface waves are neglected, so that comparison between the prediction of the model and fully-vectorial numerical results will show the respective contributions from SPPs and from other surface waves to the antenna radiation. Here we use the term “surface wave” to refer to a superposition of all waveguide modes on the antenna arms (see Supplementary Information for the definition and calculation of waveguide modes), which includes the SPP and other surface waves (the latter, also called residual field here, is a superposition of all evanescent or radiative waveguide modes on the antenna^42^).

For the dipole antenna as sketched in Fig. 1(a), we use *a*_1_, *a*_2_, *b*_1_ and *b*_2_ to denote the unknown coefficients of SPPs that propagate away from and toward the nano-gap, respectively. For solving the SPP coefficients, a set of coupled-SPP equations can be written,
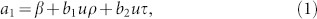

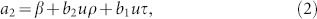

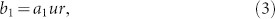

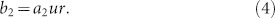
In Eqs. (1)–(4), *u* = exp(*ik*_0_*n*_eff_*L*) denotes the phase shift of the SPP accumulated over one antenna arm (*k*_0_ = 2π/*λ*, *n*_eff_ the complex effective index of the SPP mode, *k*_0_*n*_eff_ being the SPP propagation constant), *β* denotes the excitation coefficient of the SPP by the dipole source [see Fig. 1(c)], *ρ* and *τ* are the elastic reflection and transmission coefficients of the SPP at the nano-gap [Fig. 1(d)], and *r* is the reflection coefficient of the SPP mode at the antenna end^43^ [see Fig. 1(e)]. All these scattering coefficients, *β*, *ρ*, *τ*, *r*, can be calculated with the fully-vectoral a-FMM^38^ that employs a stable scattering matrix algorithm^44^ (without using any fitting process of numerical or experimental data, see Supplementary Information for some details of the calculation). The equations can be understood intuitively. For Eq. (1), the coefficient *a*_1_ of the left-going SPP on the left arm results from three contributions: the first one (*β*) from the direct excitation of the source in the nano-gap; the second one from the reflection (*ρ*) of the damped (*u*) right-going SPP (coefficient *b*_1_) that originates from the left end of the antenna; the third one due to the transmission (*τ*) of the damped (*u*) left-going SPP (*b*_2_) that originates from the right end of the antenna. The other equations can be understood in a similar way. Solving Eqs. (1)–(4), we can obtain,
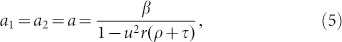

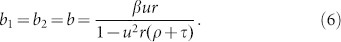
Then the electromagnetic field in the nano-gap can be expressed as,
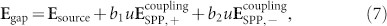
where **E**_source_ denotes the field excited by the dipole source for infinite-length antenna [see Fig. 1(c)], 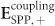
represents the field coupled from a right-going SPP into the nano-gap [Fig. 1(d)], and 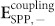 is defined similarly for a left-going SPP. **E**_source_, 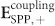 and 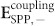 can be obtained via fully-vectorial calculations (see Supplementary Information for some details of the fully-vectorial a-FMM). Equation (7) shows that the field in the gap results from three contributions: the first contribution from a direct excitation by the source; the second and the third contributions from the coupling of two counter-propagating SPPs. Equation (7) is valid within the tiny region of the nano-gap, which is bounded by the two gap walls in *z*-direction and roughly by the transversal size of antenna arms in *x*- and *y*-directions. With Eq. (7), the total emission rate Γ_total_ = −Re[*E_z_*(0,0,0)]/2 is then obtained from the electric field *E_z_*(0,0,0) at the center of the gap where the source locates.

To calculate the radiative emission rate Γ_rad_ with the SPP model, the electromagnetic field in free space can be expressed as,
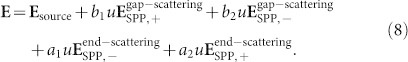
In Eq. (8), **E**_source_ denotes the field excited by the source for dipole antenna with infinite-length arms [Fig. 1(c)]. 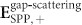 [Fig. 1(d)] and 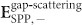 denote the field scattered by the nano-gap for an incident right-going or left-going SPP. 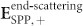 [Fig. 1(e)] and 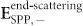 represent the field scattered by the antenna end for an incident right-going or left-going SPP. The scattered fields 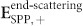 and 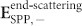 can be obtained with fully-vectorial calculations, for which the incident SPP that propagates over an infinite-length nano-wire has been removed. Equation (8) shows that the electromagnetic field radiated into free space contains five contributions: the field directly excited by the source; the two fields scattered at the gap for two in-going SPPs (with coefficients *b*_1_ and *b*_2_); and the two fields scattered at the antenna ends for two out-going SPPs (coefficients *a*_1_ and *a*_2_). With Eq. (8), Γ_rad_ is then obtained by calculating an integral of the Poynting vector over a close surface encompassing the antenna.

## Comparison between fully-vectorial numerical data and model predictions

**Figure 2:**
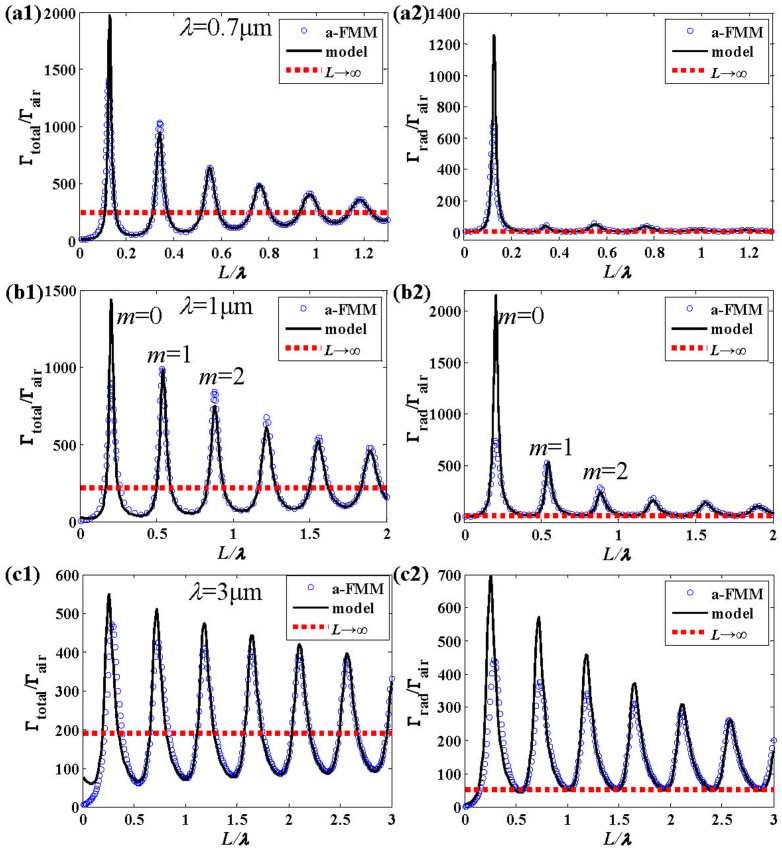
Total (Γ_total_ in the left column) and radiative (Γ_rad_ in the right column) emission rates of the dipole antenna plotted as functions of antenna length *L*. Γ_total_ and Γ_rad_ are normalized by the emission rate Γ_air_ of a dipole source in air. The horizontal dotted lines show the results for antennas with infinite-length arms (*L*→∞). The results are obtained for wavelengths *λ* = 0.7 μm (a1)–(a2), 1 μm (b1)–(b2) and 3 μm (c1)–(c2), respectively. Other parameters are *D* = 0.04 μm and *w* = 0.03 μm. The complex effective index of the SPP mode is *n*_eff_ = 2.3691 + 0.1066*i*, 1.4824 + 0.0352*i*, 1.0819 + 0.0108*i* for *λ* = 0.7 μm, 1 μm, 3 μm, respectively. (1.5 column)

We first calculate the total (Γ_total_) and radiative (Γ_rad_) emission rates for different antenna lengths *L*, which are normalized with the emission rate Γ_air_ of an electric point source in free space of air (Γ_air_ = *η*_vac_*k*_0_^2^*n_a_*/12*π*, *k*_0_ = 2π/*λ*, *n_a_* = 1 being the air refractive index, and *η*_vac_ being the wave impedance in vacuum). The results obtained with the fully-vectorial a-FMM are shown with blue circles in Fig. 2 for different wavelengths [*λ* = 0.7, 1, 3 μm for Figs. 2(a)–2(c)]. The gap width is *w* = 0.03*λ* and the side length of antenna cross section is *D* = 0.04*λ*. The ratio Γ_total_/Γ_air_ is shown in the left column of Fig. 2, and Γ_rad_/Γ_air_ is shown in the right column. Γ_total_ is larger than Γ_rad_ due to the energy dissipation by the lossy gold. It is seen that the emission rates are drastically enhanced (Γ_total_/Γ_air_ ≫ 1, Γ_rad_/Γ_air_ ≫ 1) for some specific values of the antenna length *L*. With the increase of the antenna length, the emission rates Γ_total_ and Γ_rad_ oscillate quasi-periodically and their peak values attenuate gradually. The attenuation of the peak values is slower for longer wavelengths.

Here we defined two radiation enhancement factors, *γ*_T_ = Γ_total_/Γ_air_ and *γ*_R_ = Γ_rad_/Γ_air_, which are of great importance related to practical applications. For instance, the enhancement factor *γ*_T_, also called Purcell Factor that characterizes the enhancement of the spontaneous emission rate^45^, is required to achieve high values for single-photon sources used in high-speed quantum information processing^46^. For the fluorescence sensing of molecules, the quantum yield of fluorescence signals modified by the antenna can be expressed as^17^,^47^, *η* = *γ*_R_/(*η*_0_^−1^ − 1 + *γ*_T_), where *η*_0 _represents the intrinsic quantum yield of molecules. It is seen that for molecules with a high *η*_0_, the modified quantum yield reduces to^14^,^48^ *η* ≈ *γ*_R_/*γ*_T_, which is defined as the antenna efficiency. While for molecules with a very low *η*_0_^49^,^50^, there is *η* ≈ *η*_0_*γ*_R_, proportional to the enhancement factor of the radiative emission rate.

For comparison, we also calculate the total and radiative emission rates for antennas with two semi-infinite arms [*L*→∞, sketched in Fig. 1(c)], which are denoted by Γ_total,∞_ and Γ_rad,∞_. The results are shown with the horizontal red dotted lines in Fig. 2. For the infinite-length antenna, the surface waves excited by the emitter in the nano-gap will propagate away from the gap along the antenna arms without further scattering at the antenna end or the gap, thus have no contribution to the antenna radiation. Comparison between the emission rates for finite-length and infinite-length antennas will show the impact of surface waves on the antenna radiation. Figure 2 shows that Γ_total_ can be either higher or lower than Γ_total,∞_ for different antenna lengths, which implies that surface waves on the antenna arms can either enhance or suppress the total emission rate. However, Γ_rad_ is always higher than Γ_rad,∞_ for different antenna lengths, showing that the radiative emission rate is always enhanced by the surface waves on antenna arms.

The predictions by the SPP model are shown with black-solid curves. The horizontal dotted lines in Fig. 2, which show the emission rates for infinite-length antennas (*L*→∞), correspond to the first term **E**_source_ in Eqs. (7) and (8) of the SPP model and thus exclude the contribution from surface waves on the antenna. Here we define a relative error *ε* = |Γ_model_ − Γ_a-FMM_|/Γ_a-FMM_ to quantify the deviation between model predictions and fully-vectorial data, where Γ_model_ and Γ_a-FMM_ represent the total or radiative emission rates obtained with the SPP model and the a-FMM, respectively. It is seen that at the first order of resonance [peaks at *m* = 0 defined in Eq. (9)], the prediction of the SPP model deviates from the data obtained with the fully-vectorial a-FMM. For instance, for wavelength *λ* = 1 μm, the deviations of total and radiative emission rates at resonance *m* = 0 are *ε*_total_ = 0.6042 and *ε*_rad_ = 1.9266, respectively. This evidences the contribution of surface waves other than SPPs to the antenna radiation. While at higher orders of resonance (peaks at *m* = 1, 2, …), the results obtained with the SPP model agree well with the fully-vectorial a-FMM results (for *λ* = 1 μm, *ε*_total_ = 0.0094 and *ε*_rad_ = 0.0176 at resonance *m* = 1, *ε*_total_ = 0.1087 and *ε*_rad_ = 0.1048 at resonance *m* = 2). Thus for these cases only the SPP dominantly contributes to the antenna radiation.

Equations (5)–(8) of the SPP model show that Γ_total_ and Γ_rad_ reach their peak values when |*a*| and |*b*| are maximized under a phase-matching condition,
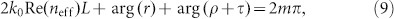
where Re(*n*_eff_) denotes the real part of the complex effective index of the SPP mode, arg() denotes the argument, and *m* is an integer corresponding to different orders of resonance. The derivation of Eq. (9) requires the second term of the denominator of Eqs. (5) and (6) be close to 1. This is achieved in view that the elastic SPP reflection and transmission coefficients (*ρ* and *τ*) are constrained by a coherent-form energy conservation relation^51^, and since very few energy is scattered by the nano-gap, |*ρ* + *τ*| is just slightly smaller than 1 (|*ρ* + *τ*| = 0.9674, 0.9072, 0.7709 at *λ* = 0.7, 1, 3 μm). |*r*| is close to 1 (|*r*| = 0.9558, 0.8837, 0.6782 at *λ* = 0.7, 1, 3 μm) due to the strong reflection of the SPP at the antenna end^43^. There is |*u*| = exp[−*k*_0_Im(*n*_eff_)*L*] ≈ 1 due to the weak damping of the propagative SPP mode over the antenna arm (*n*_eff_ = 2.3691 + 0.1066*i*, 1.4824 + 0.0352*i*, 1.0819 + 0.0108*i* for *λ* = 0.7 μm, 1 μm, 3 μm, respectively). As confirmed in Fig. 2, the antenna lengths corresponding to resonance peaks are accurately predicted by Eq. (9) with different *m* (labeled in the figure). Equation (9) shows that at resonance, the phase change accumulated by the SPP that propagates back and forth over one round on the antenna is multiples of 2*π*, which obviously results in a constructive interference of the multiple-scattered SPPs. Equation (9) predicts the location of the localized surface plasmon resonance (LSPR) considered in literatures^52^ and shows that the LSPR should result from a resonant excitation of SPPs on antenna arms (note that contribution of other surface waves should be considered at resonance *m* = 0). Under the phase-matching condition, *a* and *b* reach the resonance values of
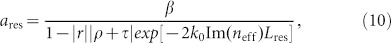

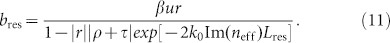
where Im(*n*_eff_) > 0 is the imaginary part of the effective index of SPP. Here *L*_res_ denotes the antenna length that fulfills the phase-matching condition of Eq. (9), and becomes larger for higher orders of resonance (i.e. larger *m*). Thus |*a*_res_| and |*b*_res_| become smaller for higher orders of resonance due to the exponential decrease in the denominator of Eqs. (10) and (11). Consequently, the enhancement factor becomes lower for higher-order resonances (as confirmed in Fig. 2). Figure 2 shows that with the increase of the antenna length, the peak values of emission rates attenuate slower for longer wavelengths, which is due to the lower propagation loss of the SPP [i.e. smaller values of Im(*n*_eff_)].

**Figure 3:**
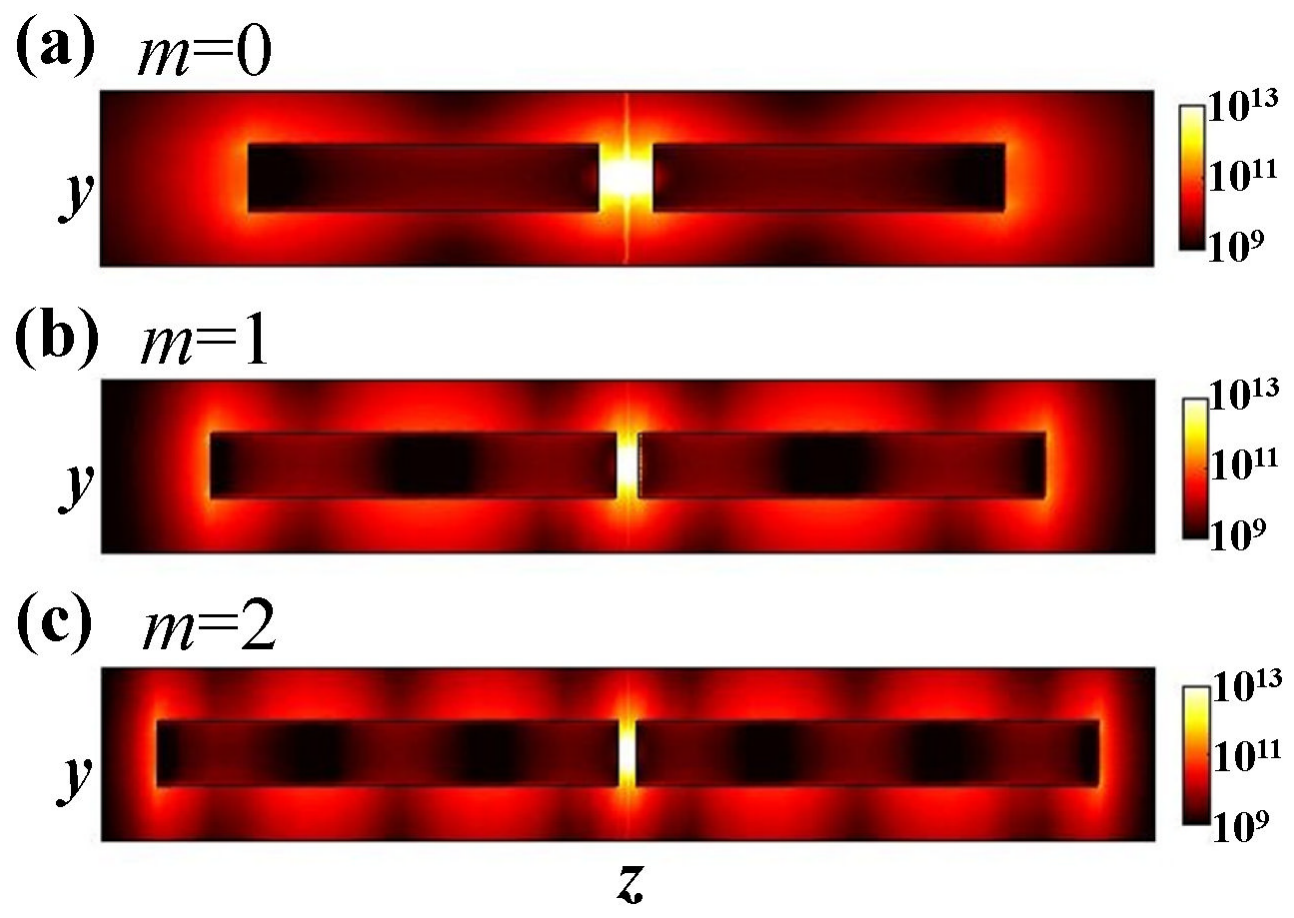
Near-field distribution (|*E_x_*|^2^ + |*E_y_*|^2^ + |*E_z_*|^2^ in SI unit) on the cross section *x* = 0 of the antenna. (a)–(c) show the results at the first three orders of resonance (*m* = 0, 1, 2), corresponding to antenna lengths *L* = 0.206 μm, 0.540 μm and 0.880 μm, respectively. The results are obtained for *λ* = 1 μm, *D* = 40nm and *w* = 30nm. (1 column)

Figures 3(a)–3(c) show the near field distribution on the antenna at the first three orders of resonance [corresponding to peaks of the emission rates in Fig. 2 obtained for *m* = 0, 1, 2 in Eq. (9) at *λ* = 1μm]. The number of nodes of field on the antenna is shown to increase with the increase of the antenna length, which results from an interference of counter-propagating surface waves on the antenna just as shown with the SPP model.

**Figure 4:**
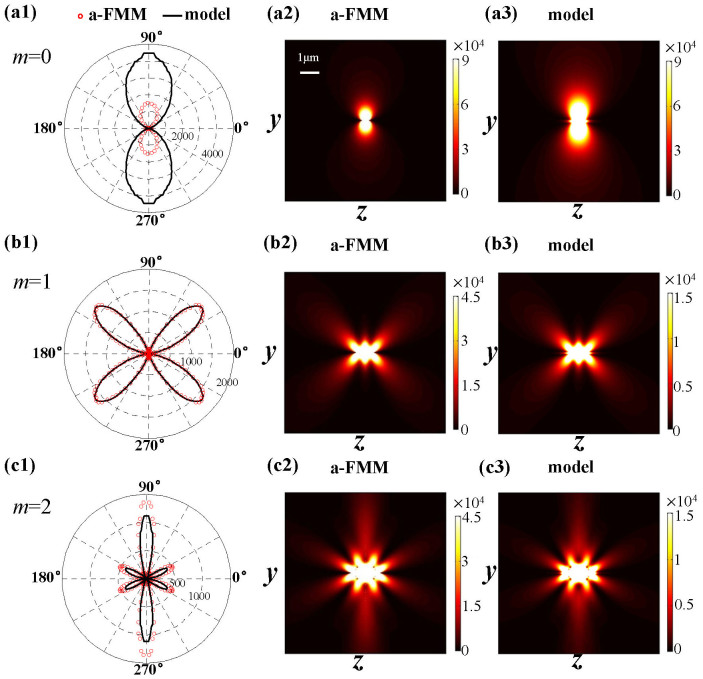
Far-field radiation pattern of the dipole antenna. The results are obtained at the first three orders of resonance, *m* = 0, 1, 2 for (a)–(c). The left column shows the angular distribution of the emitted power obtained with the fully-vectorial a-FMM (red circles) and the SPP model (black-solid curves). The middle and right columns show the modulus of the Poynting vector (in SI unit) on *x* = 0 plane obtained with the a-FMM and the SPP model, respectively. The antenna geometrical parameters are the same as those in Fig. 3. (1.5 column)

Figures 4(a)–4(c) show the far-field radiation pattern of the dipole antenna at the first three orders of resonance (*m* = 0, 1, 2 at wavelength *λ* = 1 μm). The angular distribution of the emitted power (left column in Fig. 4) is obtained by calculating the modulus of the Poynting vector (i.e. energy flux density) on a circle surrounding the antenna (in the plane *x* = 0 with a radius of 5 μm). The results (red circles obtained with the a-FMM) show that with the increase of resonance orders (or the antenna length), the number of lobes of the angular emission pattern increases. The spatial distribution of the corresponding energy flux density (in *x* = 0 plane) obtained with the fully-vectorial a-FMM is shown in the middle column in Fig. 4.

The far-field radiation pattern can also be calculated with Eq. (8) of the SPP model. The angular distribution of the emitted power obtained with the model are shown by the black-solid curves in the left column of Fig. 4 for different orders of resonance (*m* = 0, 1, 2). The corresponding distribution of energy flux density obtained with the model is shown in the right column of Fig. 4. It is seen that at the first order of resonance (*m* = 0), the SPP model well predicts the profile of the radiation pattern but exhibits deviation in predicting the absolute values of fully-vectorial a-FMM results. This shows the contribution of surface waves other than SPPs to the antenna radiation. At higher orders of resonance, the data obtained with the SPP model agree well with the fully-vectorial results, which shows the dominant role of SPPs in the antenna radiation. This is consistent with the conclusion derived from Fig. 2.

**Figure 5:**
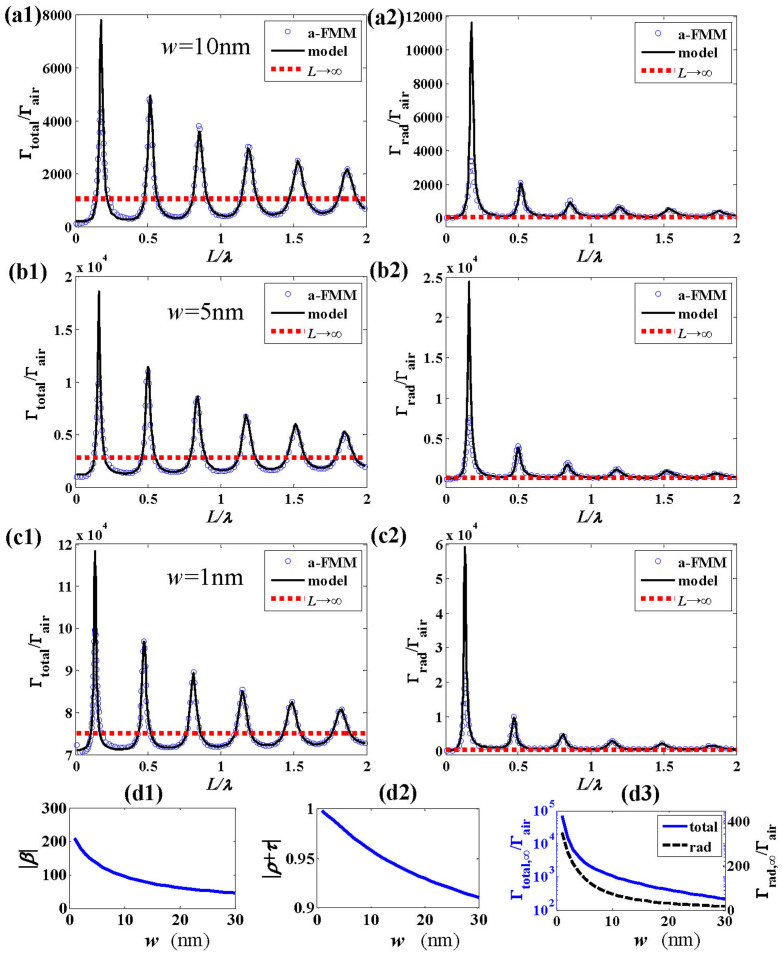
Impact of the nano-gap width *w* on the total and radiative emission rates of dipole antennas. (a)–(c) Γ_total_ and Γ_rad_ plotted as functions of antenna length *L* for *w* = 1, 5 and 10nm, respectively. The results are obtained with the fully-vectorial a-FMM (blue circles) and the SPP model (black-solid curves). The horizontal dotted lines show the results (Γ_total,∞_ and Γ_rad,∞_) for antennas with infinite-length arms (*L*→∞). (d1)–(d3) |*β*|, |*ρ* + *τ*|, Γ_total,∞_ and Γ_rad,∞_ plotted as functions of the gap width *w*. (1.5 column)

To show the impact of the width of the nano-gap on the radiation, we plot the total and radiative emission rates (Γ_total_ and Γ_rad_) as functions of the antenna length for different widths of the nano-gap. The results obtained with the fully-vectorial a-FMM are shown with blue circles in Figs. 2(b) and 5(a)–5(c) for gap widths *w* = 30, 10, 5 and 1nm, respectively (wavelength *λ* = 1 μm). The results show that the peak values of Γ_total_ and Γ_rad_ increase rapidly with the decrease of the gap width. The horizontal dotted lines represent the emission rates for dipole antennas with infinite-length arms (denoted by Γ_total,∞_ and Γ_rad,∞_ earlier, which exclude the contribution of surface waves on the antenna). It is seen that Γ_total,∞_ and Γ_rad,∞_ also increase with the decrease of the gap width. The results obtained with the SPP model are provided with the black-solid curves. Consistent with the previous results, the prediction of the SPP model is more precise at higher orders of resonance, which reveals less pronounced contribution of surface waves other than SPPs to the antenna radiation. The peak values of the emission rates are shown to be higher for narrower gaps, which should result from larger values of SPP coefficients *a* and *b* according to Eqs. (5)–(8) of the model. With Eqs. (10) and (11), the larger values of SPP coefficients *a*_res_ and *b*_res_ at resonance for narrower gaps may result from larger values of *β* and |*ρ* + *τ*| that depend on the gap width [note that *L*_res_ hardly changes with the gap width, as shown in Figs. 5(a)–5(c)]. This is confirmed by Figs. 5(d1) and 5(d2) that plot |*β*| and |*ρ* + *τ*| as functions of gap width *w*. The increase of |*ρ* + *τ*| with the decrease of gap width is due to the lower energy loss for the elastic transmission and reflection of the SPP [sketched in Fig. 1(d)] at the nano-gap^51^. The increase of |*β*| with the decrease of the gap width is related to the increase of the coupled field 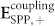 [defined in Fig. 1(d)] via the reciprocity relationship^53^. Note that the reflection coefficient *r* of the SPP at the antenna end is independent of the gap width (*r* = −0.5707 − 0.6747*i* for *λ* = 1 μm and *D* = 40nm). As shown by Fig. 5(d3) [also see the horizontal dotted lines in Figs. 5(a)–5(c)], Γ_total,∞_ and Γ_rad,∞_ that exclude the contribution from surface waves increase monotonically with the decrease of the gap width.

## Residual waves on the antenna

**Figure 6:**
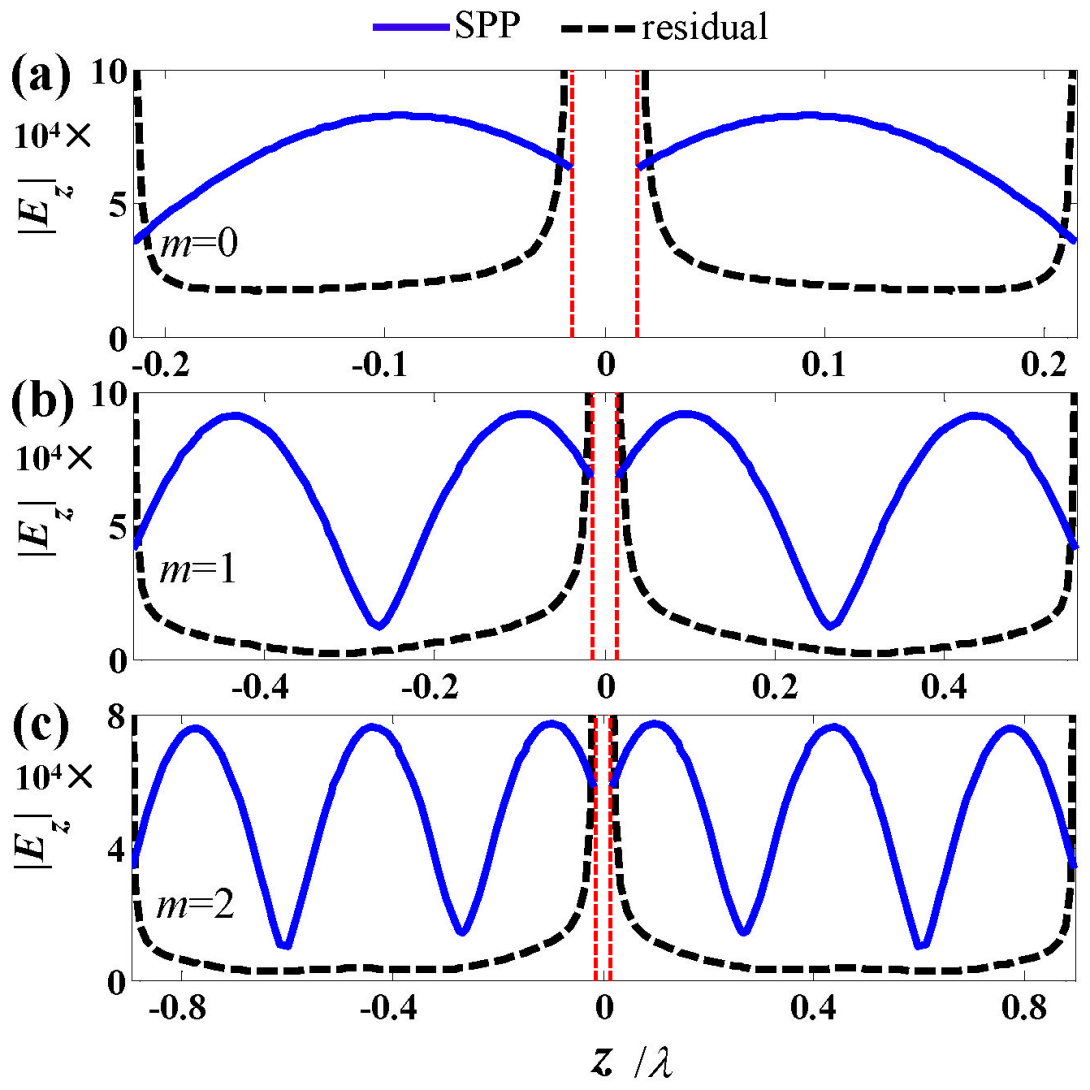
Tangential electric-field component (|*E_z_*| in SI unit) of the SPP and the residual field on the antenna surface (obtained at *x* = *D*/2, *y* = 0). (a)–(c) show the results at the first three orders of resonance (*m* = 0, 1, 2), corresponding to antenna lengths *L* = 0.206 μm, 0.540 μm and 0.880 μm for *λ* = 1 μm, respectively. The antenna geometrical parameters are the same as those in Fig. 3. (1 column).

The previous results show that for dipole antennas with short arms that support lower orders of resonance, surface waves other than SPPs have distinct contributions to the radiation of the antenna. While for dipole antennas with long arms that support higher orders of resonance, the SPP contributes dominantly to the antenna radiation. To see this directly, we show the residual field other than SPPs on the antenna at resonance. For instance, on the right arm of the dipole antenna, the SPP field is expressed as
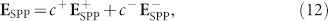
where 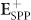 and 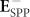 represent the electromagnetic field of two counter-propagating SPPs, and *c*^+^ and *c*^−^ are the corresponding complex coefficients that can be obtained with the fully-vectoral a-FMM^38^ [see Supplementary Information for some details of the calculation]. The residual field is then obtained by removing the SPP field from the total field on the antenna. Figures 6(a)–6(c) show the SPP field and the residual field at the surface of antenna arms, which are obtained at the first three orders of resonance [*m* = 0, 1, 2 in Eq. (9), corresponding to arm lengths *L* = 0.206 μm, 0.540 μm and 0.880 μm for *λ* = 1 μm]. It is seen that at the first order of resonance (*m* = 0), the residual field is comparable with the SPP field. Whereas, at higher orders of resonance (*m* = 1, 2), the residual field is weak relative to the SPP field. This observation is consistent with the conclusion derived from the SPP model. The residual field on the antenna arms is the analog of the quasi-cylindrical wave (QCW) on flat metallic surface^54^,^55^,^56^. The QCW has been shown decaying much faster than the SPP mode with the increase of the propagation distance^56^, and thus imposing less contribution to the multiple scattering of indentation ensembles with larger separation distances on flat metallic surface^57^. Although preliminary experimental work has been performed on the excitation and propagation properties of the residual field on the antenna^42^, further work is required to fully explore the nature of the field.

## Conclusion

We investigate the emission of an electric dipole source in the nano-gap of a metallic dipole antenna. Comparison between antennas with finite- and infinite-length arms shows that the enhancement of the antenna radiation is due to the resonance of surface waves that propagate on the antenna. To distinguish the respective contributions from the SPP and from other surface waves on the antenna to the radiation, we build up a pure-SPP model in which only the SPP is considered and all other surface waves are neglected. The model is derived by considering an intuitive picture for the dynamical launching and multiple-scattering process of SPPs on the dipole antenna. The SPP model can comprehensively reproduce the electromagnetic features of the antenna radiation (such as the total and radiative emission rates and the far-field radiation pattern). The model is self-sufficient and does not rely on any fitting process with the use of rigorous numerical or experimental data. Comparisons between the prediction of the SPP model and fully-vectorial numerical results show that for dipole antennas with long arms that support higher orders of resonance, the SPP model is highly accurate which yields the dominant role played by the SPP in the antenna radiation. Whereas, for dipole antennas with short arms that support lower orders of resonance, the SPP model exhibits distinct deviations, showing that surface waves other than SPPs have considerable contributions to the antenna radiation. This conclusion is further confirmed by a direct observation that the residual field other than SPPs on the antenna is less pronounced for antennas with longer arms. From the model we can derive a phase-matching condition that predicts the resonance of the antenna, which is related to a constructive interference of the multiple-scattered SPPs. The emission rate increases rapidly with the decrease of the width of the nano-gap, which is shown with the model due to the stronger resonance of SPPs, the higher SPP excitation efficiency and the stronger gap-only effect for antennas with infinite-length arms. At longer wavelengths, the emission rates at resonance attenuate slower with the increase of the antenna length, which is attributed to the lower SPP propagation loss with the model. The present model is helpful for clarifying the underlying physics of the radiation of resonant optical antennas and may provide recipes for an intuitive design of relevant devices. The present analysis may be extended to other forms of optical antennas, for instance, single-wire antennas^7^,^18^,^19^, split ring antennas^58^,^59^ and antenna arrays^8^,^10^. Similar analysis is possible for the reciprocal phenomenon of the electromagnetic field enhancement with optical antennas under far-field illuminations^1^,^2^,^3^,^4^.

## Author Contributions

H.J. and H.L. built up the model and performed the calculation. H.J., H.L. and Y.Z. wrote the manuscript.

## Supplementary Material

Supplementary Information supplementary information

## References

[b1] Mühlschlegel P., Eisler H. J., Martin O. J. F., Hecht B. & Pohl D. W. Resonant Optical Antennas. Science 308, 1607–1609 (2005). 15947182 10.1126/science.1111886

[b2] Liu Z. T., Li E. P., Shalaev V. M. & Kildishev A. V. Near Field Enhancement in Silver Nanoantenna-Superlens Systems. Appl. Phys. Lett. 101, 021109 (2012).

[b3] Fischer H. & Martin O. J. Polarization Sensitivity of Optical Resonant Dipole Antennas. J. Eur. Opt. Soc.-Rapid. 3, 08018 (2008).

[b4] Barnard E. S., White J. S., Chandran A. & Brongersma M. L. Spectral Properties of Plasmonic Resonator Antennas. Opt. Express 16, 16529–16537 (2008). 18852761 10.1364/oe.16.016529

[b5] Della Valle G., Sondergaard T. & Bozhevolnyi S. I. Plasmon-Polariton Nano-Strip Resonators: from Visible to Infra-red. Opt. Express 16, 6867–6876 (2008). 18545389 10.1364/oe.16.006867

[b6] Taminiau T. H., Moerland R. J., Segerink F. B., Kuipers L. & van Hulst N. F. *λ*/4 Resonance of an Optical Monopole Antenna Probed by Single Molecule Fluorescence. Nano Lett. 7, 28–33 (2007). 17212435 10.1021/nl061726h

[b7] Akimov A. V. *et al.* Generation of Single Optical Plasmons in Metallic Nanowires Coupled to Quantum Dots. Nature 450, 402–406 (2007). 18004381 10.1038/nature06230

[b8] Bakker R. M. *et al.* Enhanced Localized Fluorescence in Plamsonic Nanoantennae. Appl. Phys. Lett. 92, 043101 (2008).

[b9] Giannini V. & Sánchez-Gil J. A. Excitation and Emission Enhancement of Single Molecule Fluorescence Through Multiple Surface-Plasmon Resonances on Metal Trimer Nanoantennas. Opt. Lett. 33, 899–901 (2008). 18451932 10.1364/ol.33.000899

[b10] Bakker R. M. *et al.* Nanoantenna Array-Induced Fluorescence Enhancement and Reduced Lifetimes. New J. Phys. 10, 125022 (2008).

[b11] Azoulay J., Débarre A., Richard A. & Tchénio P. Quenching and Enhancement of Single-Molecule Fluorescence under Metallic and Dielectric Tips. Europhys. Lett. 51, 374–380 (2000).

[b12] Taminiau T. H., Stefani F. D. & van Hulst N. F. Optical Nanorod Antennas Modeled as Cavities for Dipolar Emitters: Evolution of Sub- and Super-Radiant Modes. Nano Lett. 11, 1020–1024 (2011). 21322590 10.1021/nl103828n

[b13] Farahani J. N. *et al.* Bow-Tie Optical Antenna Probes for Single-Emitter Scanning Near-Field Optical Microscopy. Nanotechnology 18, 125506 (2007).

[b14] Muskens O. L., Giannini V., Sanchez-Gil J. A. & Gomez Rivas J. Strong Enhancement of the Radiative Decay Rate of Emitters by Single Plasmonic Nanoantennas. Nano Lett. 7, 2871–2875 (2007). 17683156 10.1021/nl0715847

[b15] Bharadwaj P. & Novotny L. Spectral Dependence of Single Molecule Fluorescence Enhancement. Opt. Express 15, 14266–14274 (2007). 19550702 10.1364/oe.15.014266

[b16] Estrada L. C., AramendÝa P. F. & MartÝnez O. E. 10000 Times Volume Reduction for Fluorescence Correlation Spectroscopy Using Nano-Antennas. Opt. Express 16, 20597–20602 (2008). 19065198 10.1364/oe.16.020597

[b17] Kinkhabwala A. *et al.* Large Single-Molecule Fluorescence Enhancements Produced by a Bowtie Nanoantenna. Nat. Photonics 3, 654–657 (2009).

[b18] Lu G. W. *et al.* Single-Molecule Spontaneous Emission in the Vicinity of an Individual Gold Nanorod. J. Phys. Chem. C 115, 15822–15828 (2011).

[b19] Liaw J. W., Huang C. H., Chen B. R. & Kuo M. K. Subwavelength Fabry-Pérot Resonator a Pair of Quantum Dots Incorporated with Gold Nanorod. Nanoscale Res. Lett. 7, 1–7 (2012). 23031423 10.1186/1556-276X-7-546 PMC3541211

[b20] Zhang W. H., Fischer H., Schmid T., Zenobi R. & Martin O. J. Mode Selective Surface Enhanced Raman Spectroscopy Using Nanofabricated Plasmonic Dipole Antennas. J. Phys. Chem. C 113, 14672–14675 (2009).

[b21] Jäckel F., Kinkhabwala A. A. & Moerner W. E. Gold Bowtie Nanoantennas for Surface Enhanced Ramma Scattering under Controlled Electrochemical Potential. Chem. Phys. Lett. 446, 339–343 (2007).

[b22] Höflich K., Becker M., Leuchs G. & Christiansen S. Plasmonic Dimer Antennas for Surface Enhanced Raman Scattering. Nanotechnology 23, 185303 (2012). 22498764 10.1088/0957-4484/23/18/185303

[b23] Li M. *et al.* Plasmonic Nanorice Antenna on Triangle Nanoarray for Surface-Enhanced Raman Scattering Detection of Hepatitis B Virus DNA. Anal. Chem. 85, 2072–2078 (2013). 23320458 10.1021/ac303387a

[b24] Hanke T. *et al.* Efficient Nonlinear Light Emission of Single Gold Optical Antennas Driven by Few-Cycle Near-Infrared Pulses. Phys. Rev. Lett. 103, 257404 (2009). 20366283 10.1103/PhysRevLett.103.257404

[b25] Cai W. S., Vasudev A. P. & Brongersma M. L. Electrically Controlled Nonlinear Generation of Light with Plasmonics. Science 333, 1720–1723 (2011). 21940887 10.1126/science.1207858

[b26] Novotny L. & Van Hulst N. Antennas for Light. Nat. Photonics 5, 83–90 (2011).

[b27] Harutyunyan H., Volpe G., Quidant R. & Novotny L. Enhancing the Nonlinear Optical Response Using Multifrequency Gold-Nanowire Antennas. Phys. Rev. Lett. 108, 217403 (2012). 23003302 10.1103/PhysRevLett.108.217403

[b28] Kinkhabwala A. A., Yu Z. F., Fan S. H. & Moerner W. E. Fluorescence Correlation Spectroscopy at High Concentrations Using Gold Bowtie Nanoantennas. Chem. Phys. 406, 3–8 (2012).

[b29] Huang C. P., Yin X. G., Huang H. & Zhu Y. Y. Study of Plasmon Resonance in a Gold Nanorod With an LC Circuit Model. Opt. Express 17, 6407–6413 (2009). 19365465 10.1364/oe.17.006407

[b30] Zhu D., Bosman M. & Yang J. K. A Circuit Model for Plasmonic Resonators. Opt. Express 22, 9809–9819 (2014). 24787866 10.1364/OE.22.009809

[b31] Huang J. S., Feichtner T., Biagioni P. & Hecht B. Impedance Matching and Emission Properties of Nanoantennas in an Optical Nanocircuit. Nano Lett. 9, 1897–1902 (2009). 19338279 10.1021/nl803902t

[b32] Alù A. & Engheta N. Input Impedance Nanocircuit Loading and Radiation Tuning of Optical Nanoantennas. Phys. Rev. Lett. 101, 043901 (2008). 18764328 10.1103/PhysRevLett.101.043901

[b33] Locatelli A. *et al.* Modeling of Enhanced Field Confinement and Scattering by Optical Wire Antennas. Opt. Express 17, 16792–16800 (2009). 19770896 10.1364/OE.17.016792

[b34] Mousavi S. S., Berini P. & McNamara D. Periodic Plasmonic Nanoantennas in a Piecewise Homogeneous Background. Opt. Express 20, 18044–18065 (2012). 23038352 10.1364/OE.20.018044

[b35] de Arquer F. G., Volski V., Verellen N., Vandenbosch G. A. & Moshchalkov V. V. Engineering the Input Impedance of Optical Nano Dipole Antennas Materials, Geometry and Excitation Effect. IEEE T. Antenn. Propag. 59, 3144–3153 (2011).

[b36] Ding W. *et al.* Understanding Near Far-Field Engineering of Optical Dimer Antennas Through Geometry Modification. Opt. Express 17, 21228–21239 (2009). 19997362 10.1364/OE.17.021228

[b37] Palik E. D. Handbook of Optical Constants of Solids Part II. (Academic, Orlando, 1985).

[b38] Hugonin J. P. & Lalanne P. Perfectly Matched Layers as Nonlinear Coordinate Transforms a Generalized Formalization. J. Opt. Soc. Am. A 22, 1844–1849 (2005). 10.1364/josaa.22.001844 16211811

[b39] Moharam M. G., Grann E. B., Pommet D. A. & Gaylord T. K. Formulation for Stable and Efficient Implementation of the Rigorous Coupled Wave Analysis of Binary Gratings. J. Opt. Soc. Am. A 12, 1068–1076 (1995).

[b40] Li L. F. New formulation of The Fourier Modal Method for Crossed Surface-Relief Gratings. J. Opt. Soc. Am. A 14, 2758–2767 (1997).

[b41] Chang D. E., Sørensen A. S., Hemmer P. R. & Lukin M. D. Strong Coupling of Single Emitters to Surface Plasmons. Phys. Rev. B 76, 035420 (2007).

[b42] Liu A. P. *et al.* Independently Analyzing Different Surface Plasmon Polariton Modes on Silver Nanowire. Opt. Express 22, 23372–23378(2014). 25321806 10.1364/OE.22.023372

[b43] Gordon R. Reflection of Cylindrical Surface Waves. Opt. Express 17, 18621–18629 (2009). 20372593 10.1364/OE.17.018621

[b44] Li L. F. Formulation and Comparison of Two Recursive Matrix Algorithms for Modeling Layered Diffraction Gratings. J. Opt. Soc. Am. A 13, 1024–1035 (1996).

[b45] Purcell E. M. Proceeding of the American Physics Society. Phys. Rev 69, 681 (1946).

[b46] Knill E., Laflamme R. & Milburn G. J. A Scheme for Efficient Quantum Computation with Linear Optics. Nature 409, 46 (2001). 11343107 10.1038/35051009

[b47] Agio M. Optical Antennas as Nanoscale Resonators. Nanoscale 4, 692–706 (2012). 22175063 10.1039/c1nr11116g

[b48] Anger P., Bharadwaj P. & Novotny L. Enhancement And Quenching of Single-Molecule Fluorescence. Phys. Rev. Lett. 96, 113002 (2006). 16605818 10.1103/PhysRevLett.96.113002

[b49] Tam F., Goodrich G. P., Johnson B. R. & Halas N. J. Plasmonic Enhancement of Molecular Fluorescence. Nano Lett. 7, 496–501 (2007). 10.1021/nl062901x 17256995

[b50] Biteen J. S., Lewis N. S., Atwater H. A., Mertens H. & Polman A. Spectral Tuning of Plasmon-Enhanced Silicon Quantum Dot Luminescence. Appl. Phys. Lett. 88, 131109 (2006). 10.1021/nl061494m 17090102

[b51] Liu H. T. Coherent-Form Energy Conservation Relation for the Elastic Scattering of a Guided Mode in a Symmetric Scattering System. Opt. Express 21, 24093–24098 (2013). 24104318 10.1364/OE.21.024093

[b52] Huang J. S. *et al.* Mode Imaging and Selection in Strongly Coupled Nanoantennas. Nano Lett. 10, 2105–2110 (2010). 10.1021/nl100614p 20411912

[b53] Vassallo C. Optical Waveguide Concepts. (Elsevier, Amsterdam, 1991).

[b54] Liu H. T. & Lalanne P. Microscopic Theory of The Extraordinary Optical Transmission. Nature 452, 728–731 (2008). 18401405 10.1038/nature06762

[b55] Liu H. T. & Lalanne P. Light Scattering by Metallic Surfaces With Subwavelength Patterns. Phys. Rev. B 82, 115418 (2010).

[b56] Lalanne P., Hugonin J. P., Liu H. T. & Wang B. A Microscopic View of The Electromagnetic Properties of Sub-λ Metallic Surfaces. Surf. Sci. Rep. 64, 453–469 (2009).

[b57] van Beijnum F. *et al.* Quasi-Cylindrical Wave Contribution in Experiments on Extraordinary Optical Transmission. Nature 492, 411–414 (2012). 23257884 10.1038/nature11669

[b58] Aydin K. *et al.* Split-Ring-Resonator-Coupled Enhanced Transmission through a Single Subwavelength Aperture. Phys. Rev. Lett. 102, 013904 (2009). 19257195 10.1103/PhysRevLett.102.013904

[b59] Yang Y., Dai H. T. & Sun X. W. Split Ring Aperture for Optical Magnetic Field Enhancement by Radially Polarized Beam. Opt. Express 21, 6845–6850 (2013). 23546066 10.1364/OE.21.006845

