# Supplementary material for: Role of surface plasmon polaritons and other waves in the radiation of resonant optical dipole antennas
Source: Sci Rep. 2015 Feb 13;5:8456. doi: 10.1038/srep08456 (PMC4326694; doi:10.1038/srep08456)
Supplement: Supplementary Information [file srep08456-s1.pdf]

Role of surface plasmon polaritons and other waves in the radiation of resonant optical dipole antennas (supplementary information)

Hongwei Jia,[1] Haitao Liu,[1,*] and Ying Zhong[2]

[1]*Key Laboratory of Optical Information Science and Technology, Ministry of Education, Institute of Modern Optics, Nankai University, Tianjin 300071, China*

[2]*State Key Laboratory of Precision Measuring Technology and Instruments, Tianjin University, Tianjin 300072, China*

**Email: liuht@nankai.edu.cn*

Here we provide some details on the fully-vectorial aperiodic Fourier modal method (a-FMM)[1] [the calculation is performed with an in-house software, Liu H.T., DIF code for modeling light diffraction in nanostructures (Nankai University, China, 2010)] that is used for calculating the electromagnetic field, the scattering coefficients in the SPP model and the residual field on the antenna arms (shown in Fig. 6).

The a-FMM is a generalized version of the rigorous coupled wave analysis (RCWA) by incorporating perfectly matched layers (PMLs). The PML satisfies outgoing-wave conditions in the transversal x- and y-directions and is introduced as a nonlinear coordinate transform[1], $x=F_x(x')$ and $y=F_y(y')$. With the new coordinates, the infinite transversal range is mapped into a finite supercell, $x'\in[-\Lambda_x/2,\Lambda_x/2]$ and $y'\in[-\Lambda_y/2,\Lambda_y/2]$. Then by building up an artificial periodicity of the supercell (periods Λ_x and Λ_y), the Maxwell's equations are then reformulated to be periodic systems and can be solved with the algorithm of RCWA[2,3]. In RCWA, any component of the electromagnetic field is expanded upon the Fourier basis with respect to the new coordinates x' and y'. With the expansion substituted into frequency-domain Maxwell's equations, the resultant system of linear ordinary differential equations for the unknown Fourier coefficients (dependent on z) is then integrated analytically in each z-invariant layer, which expresses electromagnetic field in terms of z-propagating waveguide modes[2-4]. For instance, the E_x component of electric field in each z-invariant layer can be expressed as

$$E_x(x',y',z)=\sum_{m=-M}^{M}\sum_{n=-N}^{N}S_{m,n}(z)\exp(imK_x x')\exp(inK_y y'), \tag{S1}$$

$$S_{m,n}=\sum_{p=1}^{P}w_{m,n,p}\{c_p^+\exp(i\beta_p z)+c_p^-\exp[-i\beta_p(z-d)]\}, \tag{S2}$$

where M and N are the truncated harmonic numbers of Fourier series, $K_x = 2\pi/\Lambda_x$ and $K_y=2\pi/\Lambda_y$. β_p is the eigenvalue of the coefficient matrix of the linear ordinary differential equations derived from the RCWA, and corresponds to the propagation constant of the waveguide modes. $w_{m,n,p}$ is the element of the corresponding eigenvector. z is the local z-coordinate in each z-invariant layer, and d is the thickness of the layer. c_p^+ and c_p^- are the coefficients of the forward-propagating and backward-propagating waveguide modes, and are determined with the continuity boundary condition of tangential electromagnetic field at interfaces between adjacent z-invariant layers. By

changing the summation order of Eqs. (S1) and (S2), the field in each z-invariant layer is then expressed in terms of waveguide modes,

$$E_x(x',y',z)=\sum_{p=1}^{P}c_p^+E_{p,x}^+(x',y',z)+\sum_{p=1}^{P}c_p^-E_{p,x}^-(x',y',z)\,, \tag{S3}$$

$$E_{p,x}^+(x',y',z)=\sum_{m=-M}^{M}\sum_{n=-N}^{N}w_{m,n,p}\exp(imK_xx')\exp(inK_yy')\exp(i\beta_pz)\,, \tag{S4}$$

$$E_{p,x}^-(x',y',z)=\sum_{m=-M}^{M}\sum_{n=-N}^{N}w_{m,n,p}\exp(imK_xx')\exp(inK_yy')\exp[-i\beta_p(z-d)]\,, \tag{S5}$$

where Eqs. (S4) and (S5) give the expression of forward-propagating and backward-propagating waveguide modes.

For the dipole antenna shown in Fig. 1(a), the whole space is divided into six z-invariant layers, the two semi-infinite air layers on the left and right sides, the two arms of the dipole antenna, and the two air layers in the gap separated by the source. These z-invariant layers are separated by five transversal interfaces. The source is treated as a discontinuity of tangential components of electromagnetic field at the interface between the two air layers in the gap. A stable scattering-matrix propagation algorithm[5] is adopted for matching the boundary condition to determine the waveguide mode coefficients c_p^+ and c_p^- in Eqs. (S2) or (S3).

To obtain the quantities in the SPP model, the SPP mode is the fundamental waveguide mode in either z-invariant antenna-arm layer, which corresponds to a specific term in Eq. (S3) (with propagation constant $\beta_p=k_0n_{\text{eff}}$). The field distribution of the SPP mode in the transversal x- and y-directions can be calculated with Eq. (S4) by setting z=0 [see the results in Fig. (1b)]. The SPP reflection and transmission coefficients ρ and τ illustrated in Fig. 1(d) can be obtained from the corresponding coefficients c_p^+ and c_p^- of the SPPs reflected and transmitted on the left and right semi-infinite antenna arms. The SPP reflection coefficient r [Fig. 1(e)] and excitation coefficient β [Fig. 1(c)] can be obtained similarly. All the scattering coefficients β, ρ, τ and r are obtained with rigorous numerical calculations and do not depend on the length of antenna arms.

For the calculation of the SPP field and the residual field shown in Fig. 6, the SPP field on the two antenna arms is obtained from the SPP terms in Eq. (S3). The residual field is then obtained by removing the SPP terms on the right side of Eq. (S3). Thus the SPP field shown in Fig. 6 is obtained with fully-vectorial a-FMM instead of the SPP model, which contains two contributions: the first contribution predicted by the SPP model and the second contribution excited by the residual field on the antenna arms. So if the residual field is weak (for instance, for dipole antennas with long arms that support higher-order resonances), the SPP field obtained with the a-FMM should be approximately equal to that obtained with the SPP model [i.e. for Eqs. (5), (6) and (12), the SPP coefficients $c^+\approx a$ and $c^-\approx b$]. This is confirmed by the results in Fig. S1, which show that $c^+\approx a$ and $c^-\approx b$ at higher-order resonances ($m\geq1$) and deviation appears at resonance m=0.

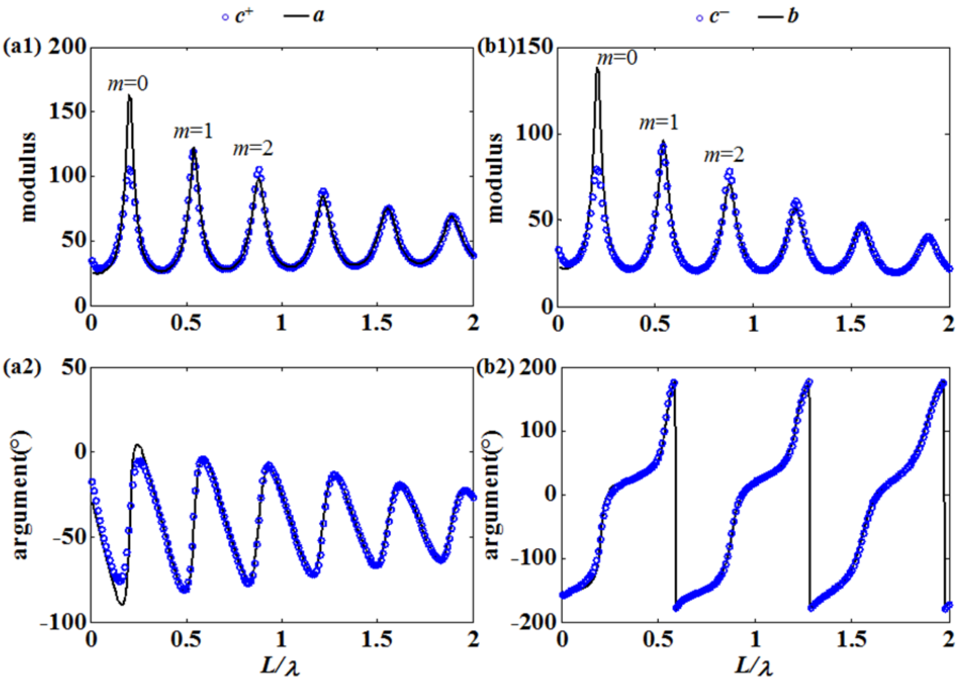

FIG. S1. SPP mode coefficients on the right antenna arm plotted as functions of the antenna length *L*. (a1)-(a2) Modulus and argument of c^+ (obtained with fully-vectorial a-FMM) and *a* (obtained with the SPP model) for forward-going SPP. (b1)-(b2) Modulus and argument of c^- (a-FMM) and *b* (SPP model) for backward-going SPP. The antenna geometrical parameters are the same as those in Fig. (b1) in the main text.

References

1. Hugonin, J.P. & Lalanne, P. Perfectly Matched Layers as Nonlinear Coordinate Transforms a Generalized Formalization. *J. Opt. Soc. Am. A* **22**, 1844-1849 (2005).

2. Moharam, M.G., Grann, E.B., Pommet, D.A. & Gaylord, T.K. Formulation for Stable and Efficient Implementation of the Rigorous Coupled Wave Analysis of Binary Gratings. *J. Opt. Soc. Am. A* **12**, 1068-1076 (1995).

3. Li, L.F. New formulation of The Fourier Modal Method for Crossed Surface-Relief Gratings. *J. Opt. Soc. Am. A* **14**, 2758-2767 (1997).

4. Vassallo, C. Optical Waveguide Concepts. Elsevier (1991).

5. Li, L.F. Formulation and Comparison of Two Recursive Matrix Algorithms for Modeling Layered Diffraction Gratings. J. Opt. Soc. Am. A 13, 1024-1035 (1996).
